# Orofacial Pain and Dentistry Management: Guidelines for a More Comprehensive Evidence-Based Approach

**DOI:** 10.3390/diagnostics13172854

**Published:** 2023-09-04

**Authors:** Mauro Labanca, Marzia Gianò, Caterina Franco, Rita Rezzani

**Affiliations:** 1 Anatomy and Physiopathology Division, Department of Clinical and Experimental Sciences, University of Brescia, 25123 Brescia, Italy; marzia.giano@unibs.it (M.G.); c.franco@unibs.it (C.F.); rita.rezzani@unibs.it (R.R.); 2 Italian Society for the Study of Orofacial Pain (Società Italiana Studio Dolore Orofacciale—SISDO), 25123 Brescia, Italy; 3 Interdipartimental University Center of Research “Adaption and Regeneration of Tissues and Organs (ARTO)”, University of Brescia, 25123 Brescia, Italy

**Keywords:** orofacial and odontogenic pain, health problem, pain management, overtreatment and mistreatment

## Abstract

Orofacial pain represents one of the most common health problems that negatively affects the activities of daily living. However, the mechanisms underlying these conditions are still unclear, and their comprehensive management is often lacking. Moreover, even if pain is a common symptom in dentistry, differential diagnostic procedures are needed to exclude other pain origins. Misinterpretation of the pain origin, in fact, can lead to misdiagnosis and to subsequent mismanagement. Pain in the orofacial area is the most common reason for patients to visit the dentist, but this area is complex, and the pain could be associated with the hard and soft tissues of the head, face, oral cavity, or to a dysfunction of the nervous system. Considering that the origins of orofacial pain can be many and varied, a thorough assessment of the situation is necessary to enable the most appropriate diagnostic pathway to be followed to achieve optimal clinical and therapeutic management.

## 1. Introduction

Orofacial pain represents one of the most widespread health problems that negatively affects daily living activities [1]. Considering the entire orofacial district, a good percentage of patients complain of pain mainly localized at the oral/dental site: the background of orofacial pain, indeed, is most associated to disease of the teeth. For this reason, we can identify it as odontogenic pain [2].

In general, the most common causes of dental pain are represented by dental caries or tooth decay. Moreover, when untreated, even a simple dental caries can become a more generalized oral issue, leading to the second group of conditions most associated with dental and orofacial pain: a general inflammation of the dental pulp known as pulpitis [1,2,3,4,5,6,7,8]. Another common odontogenic pain condition is represented by a short and sharp pain that occurs in response to various exogenous stimuli and that cannot be ascribed to any other organic causes—a condition known as dentine hypersensitivity [1,8,9,10].

It is possible to say that any external stimulus, if it is directed to the exposed dentine or tooth with pulpitis, can cause a painful sensation.

**Figure 1:**
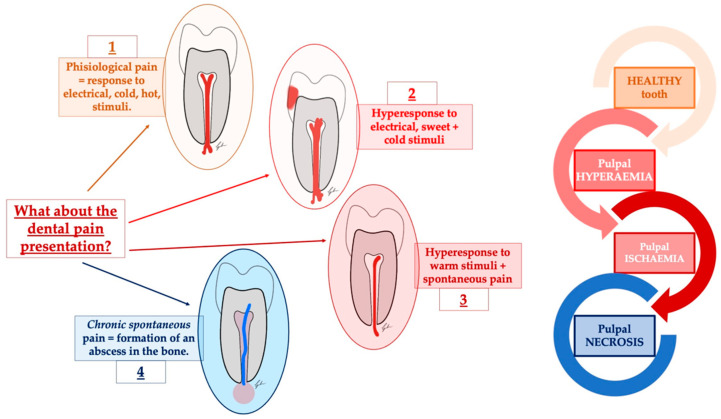
A schematic representation of the many guises of dental pain. In dental pain and toothache, many features can be described. Among them, the most common are represented by the elicited pain that characterizes the form of reversible pulpitis, in which the response to touch and cold can mimic trigeminal neuralgia, post-traumatic neuropathies, trigeminal autonomic cephalalgias, and other secondary neuropathies. In the end, the episodic intense throbbing pain associated with irreversible pulpitis can mimic myofascial pain and migraine.

However, most importantly, what we have to consider is the fact that the sensory system and the innervation in the tooth and, in general, in the orofacial district, are unique and complex (Figure 1). Therefore, even if many of the dental conditions are well-known and well-defined, it is not the same for the transduction of the nociceptive signal and, consequently, for the treatment [11,12]. Some of these underlying mechanisms, in fact, remain unclear, and this could lead to undertreatment of the oral pain, to overtreatment even in cases where intervention could be avoided, or, ultimately, to inappropriate treatment [1].

In addition, we have to consider that lesions or diseases that involve the somatosensory nervous system may paradoxically not only lead to a loss of function but also to increased pain sensitivity and spontaneous pain.

The point is exactly the one that Pigg and colleagues highlighted in their recent work: “Misinterpretation of the pain origin can lead to misdiagnosis and to subsequent mismanagement” [13]. This concept is true and valid in a general meaning in the medical field, but it takes on even greater significance in the dental field. Moreover, inadequately diagnosed, or managed, pain in dentistry is the most common adverse event reported by both dentists and patients [11,14]. In this context, interpreting the patient’s signs and symptoms correctly, giving them the right attention, and, above all, the right weight, becomes fundamental.

Considering that the origins of orofacial pain may be diverse, the aim of the authors of this review is to summarize the main features that characterize general, acute, and chronic orofacial pain, with a focus on neuropathic pain and its treatment. Furthermore, we would underline the principal aspects that have to be considered in the management of chronic orofacial pain, highlighting some of the points that could help in making a proper diagnosis and, in addition, to guide dentists to make a differential diagnosis between neuropathic and neuralgic pain [8].

## 2. What Is Pain?

Considering what has been said up to now, before talking about pain management, it is important to have a clear definition of what pain is.

The neurologist George Riddoch, in his works from the first half of the twentieth century, talks about pain and gives the following definition: (it) “is experienced only intermittently in the life of the healthy, its neural mechanisms lying dormant, but vigilant, ready to be awakened if the tissues of the body are threatened” [15,16].

From this first description, pain can be considered a sort of “tool”, used by our organism as self-defense. In fact, according to this definition, even healthy individuals during their life can experience, for a limited period of time, pain. And for this reason, pain has been defined, by some authors, as a specific form of sensitivity, a kind of “sixth sense” [16,17,18,19].

Human beings have experienced pain since ancient times as works of art, as well as historical and literary evidence, can confirm. And this has led to an ever-increasing scientific interest in this field. From Galenus to Doctor Bonica—who is considered the father of pain medicine—the amount of knowledge that is available to us about pain and how to define it has increased considerably. In 1979, in fact, Merskey H. gave a second definition of pain, in which he tried not only to describe the sensation but also to find a correlation between it and its origin [20,21]. Pain has been described as “an unpleasant sensory and emotional experience associated with actual or potential tissue damage, or terms of such damage”. This definition of pain, currently used also by the IASP (International Association for the Study of Pain), is important because it has enabled us to understand that there is a first type of pain, that can be considered a physiological pain, that differs from that form of pain that is no longer a reflection of tissue damage but more related to the psychological aspect [8,22].

### 2.1. Definition and Classification of Pain

Our sensory system is the part of the nervous system responsible for the perception of changes and modifications that take place. Considering the perception of a painful stimulus, we can speak about nociception, which refers to the combination of all the neural feedbacks and pathways that the central nervous system (CNS) carries out to detect and prevent dangerous and/or potentially damaging stimuli. The process of nerve transduction begins with the receptors, structures in our bodies that allow us to establish a connection with the environment (both internal and external) [23,24]. The role of the CNS, then, is to convert all the environmental stimuli into electrochemical signals that are able to evoke an electric potential, which is then converted in the brain at the level of the cortex into the actual perception of pain [11,25,26].

The sensation of pain differs from the other specific senses that our body can experience. In fact, it is not simply a one-way transmission of one input through a direct pain pathway [27,28,29,30]. On the contrary, many potential inputs and outputs along with many biological systems and regions of the CNS are involved. Moreover, each of these steps can be influenced by many internal and external factors.

Knowing that the pain transmission works in a different way is important for shaping the correct therapeutical strategy [30,31,32,33].

According to this first distinction, the IASP defines three main pain mechanisms: nociceptive pain, neuropathic pain, and nociplastic pain. In general, we can speak about nociceptive pain as a pain that comes from a recognizable damage (actual or potential). The damage in this case is localized in a non-neural tissue, and the pain transmission starts with the activation of nociceptors. Differently, neuropathic pain is a pain that can be associated with a lesion, a damage, or a disease of the somatosensory nervous system: in this case it is the nervous system that is directly involved not only in the transmission but also by the direct damage. There is also a new category named “nociplastic pain”: it is a type of pain that comes from an altered nociception. In this case, it is not possible to clearly identify actual or potential tissue damage leading to the activation of peripheral nociceptors. Moreover, at the same time, it is not possible to highlight the presence of a disease or lesion directly affecting the somatosensory system [30].

**Figure 2:**
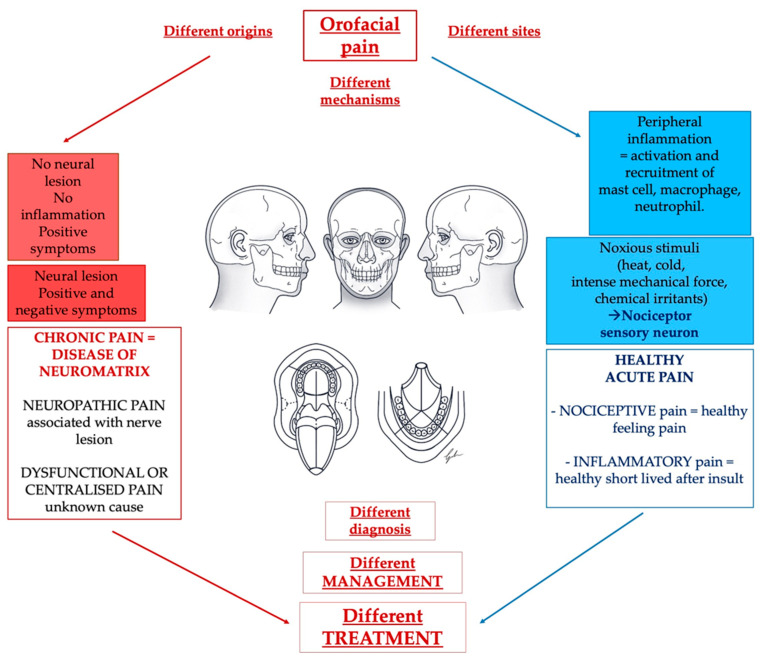
A challenge for the dentist regarding pain management is represented by the multitude of causes and situations that can lead to the presentation of orofacial pain. We can highlight healthy nociceptive and inflammatory pain next to an unhealthy non-protective pain that is mainly represented by neuropathic pain with or without autonomic components and dysfunctional or centralized pain.

Focusing on orofacial pain, in 2020, the International Classification of Orofacial Pain (ICOP) introduced a specific classification to better describe orofacial pain [34]. Over the years, a detailed classification of orofacial pain has become increasingly necessary. This is because scientific and technological advances have made available new tools for diagnosis and new treatment protocols that have led to increasingly specialized and customized precision medicine (Figure 2).

### 2.2. Acute and Chronic Pain

As mentioned before, the neural transmission begins with an external pain stimulus that is recognized by a receptor. This normally leads to a self-limiting sensation of pain, which, simplifying, can be identified as a form of acute pain [25,26].

This first stimulus can then be associated with the release of pro-inflammatory markers by the nociceptive neurons: this leads to nociceptive pain with a typical inflammatory pattern. The release of these pro-inflammatory markers can modify the perception of the initial pain stimulus by going on to increase local sensitivity stimulating or activating responsive cells in their local environment. Moreover, the release of the nociceptor-induced inflammatory markers leads to an expansion of the pain stimulus that no longer remains localized at the point initially involved by the stimulus but may become enlarged, occurring over an area greater than that of the original nociceptor(s) [25,26]. Both the initial pain transmission and the associated inflammatory pattern normally resolve spontaneously quite quickly. Alongside what we have just described, however, there is chronic pain. It affects more than 10% of adults in the general population. The concept of chronic pain reflects what was mentioned earlier: even if pain is an important acute warning signal that allows the organism to react to a potential or dangerous condition, if the sensation does not disappear, it can become persistent and lose its initial protective role [35].

According to a general definition, pain can be depicted as persistent, or chronic, when it lasts for more than 3 months.

One of the key points that has to be considered in this case is the social and emotional dimensions that affect the patient with chronic pain. Also, the treatment plan must be accorded to the duration of pain; what can be successfully used for dealing with acute pain cannot be used, evidently, for chronic pain. Over time, several models have been developed to obtain a more in-depth study of pain. Among the options, the one that is most often considered is an integrative model [35,36]. In it, pain could be explained by combining a motivation-decision model, a fear-avoidance model, adding the learned helplessness, and, in the end, a Bayesian expectation integration model [35,37,38].

## 3. Orofacial Pain

Orofacial pain is defined as pain that originates primarily from the regions of the face and mouth [10]. The orofacial region is a very complex region from an anatomical point of view. It houses an enormous breadth of structures, and each of them has very complex vasculature and equally complicated innervation [39].

In general, it is possible to assume that in the orofacial area, dental pain is the most common type of pain. And, in most cases, it is an inflammatory pain [10]. However, as mentioned before, more or less any external stimulus (even one which is not specifically painful) in this area can trigger a sensation of pain [40,41,42].

As the starting point, it is possible to consider the complex innervation that this area presents. This is also the reason why most patients presenting pain are almost never able to locate it in a specific and limited area. This explains why most cases of orofacial pain end up at the attention of the dentist even though, very often, the pain stimulus does not involve structures that are part of the odontostomatognathic system.

Moreover, the head and neck regions are the most common sites of the human body to be involved in chronic pain conditions [21,43,44], making it even more necessary to correctly frame the patient.

For this reason, the International Classification of Orofacial Pain (ICOP) has become an important landmark in clinical practice. It provides a useful tool in the differential diagnosis that the dentist must be able to make to identify the conditions that really require dental intervention and not another type of medical/surgical intervention [13]. The ICOP, in fact, provides a description of the different pain conditions that could be diagnosed in the orofacial region and gives some diagnostic criteria for identifying the depicted conditions. [45].

**Figure 3:**
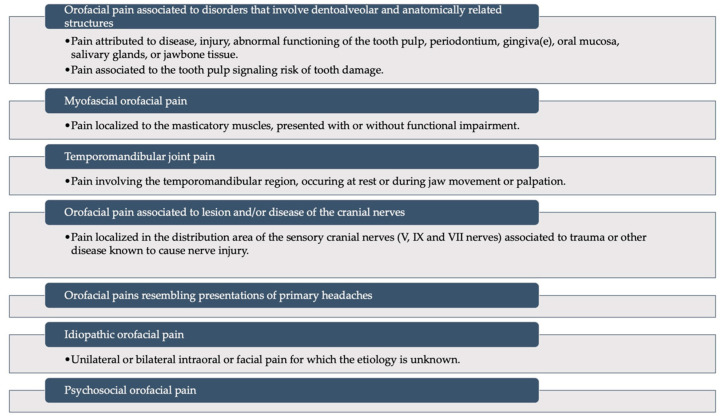
A schematic representation of the International Classification of Orofacial Pain: it recognizes six main categories of disorders that can involve the orofacial district. Modified from Pigg et al., 2021 [13] (This is an open access article distributed under the term of the Creative Commons CC-BY license).

The purpose of this review is not to go into detail about all the disorders that can affect the orofacial area, so we will not dwell on that. For completeness, we will report the list provided by ICOP to illustrate what the major “families” of disorders are that can affect and involve the orofacial area (Figure 3) [13].

### Orofacial Pain and Differential Diagnosis

The studies around the diagnosis and therapy of musculoskeletal and neuropathic diseases of the orofacial system are many and have increased over time. Pain, in fact, is a major issue in modern society [2]. Approximately 10% to 20% of individuals, in the Western part of the world, experience persistent pain and, moreover, the most important thing is that, even if the research and the interest around the diagnosis and the treatment of the pain are increased, pain outcomes have improved only a little [30,46,47,48].

Next to dental caries and periodontal diseases, musculoskeletal and neuropathological diseases are the most common cause of orofacial pain, and it is important to define a boundary because these types of pain cannot be resolved by dental intervention [2,49].

Over the years, new features have been described for each pathology, and this helped in identifying the diagnostic procedure to be followed.

Magnetic resonance imaging (MRI), for example, is the gold standard for identifying temporomandibular joint disease. It is useful because, through it, it is possible to analyze the soft tissues that in most of the cases are not clearly observable with other radiological methods [2,50].

On the other side we can find the neuropathic pain. As already mentioned before, it is initiated or caused by a lesion of the peripheral or/and central nervous system manifesting with sensory symptoms and signs. Trigeminal neuralgia is one of the most common forms of neuropathic pain, followed by a form of persistent pain. Also, in these cases, the most used radiological method of investigation is represented by the MRI.

In dental practice, it is most important to differentiate whether pain is odontogenic since the diagnostic procedures and the therapeutical procedures are different. In any case, dental pain is an extremely common symptom, and it can also co-exist with other conditions [2,51,52]. In this context, the history of the patient and the time dedicated to him is important in order to finalize a correct diagnosis. The dentistry should focus on:

1. Timing of the pain: onset, duration, and periodicity.
2. Location and irradiation (e.g., within nerve distribution) of the pain.
3. Quality and severity of the pain.
4. Relieving and aggravating factors for the pain sensation.
5. Pain’s associated factors.
6. Other pain conditions (e.g., headaches, migraines, chronic widespread pain, and fibromyalgia).
7. Overall impact of pain on the daily life.

In the same way, also the genetic and drug history of the patient should be considered in order to address in the correct way the interpretation of what has been referred from the patient.

Moreover, the examination of the patient is a fundamental step: apart from the oral cavity, an extraoral examination must be carried out also, even if it is confined generally to the head and neck region. Visual inspection will show any color changes, swellings, and skin lesions [2]. In the end, despite all the precautions, differential diagnosis is a crucial point in the dentist’s work, and misunderstanding a correct diagnosis is an easy mistake that can be made.

Additionally, for neuropathic pain, it is important to define the localization that we want to consider: the glossopharyngeal neuralgia, for example, has the same characteristics as trigeminal neuralgia except for location.

Trigeminal autonomic cephalgia represents a group of unilateral episodic pains, some of which can easily be mistaken for trigeminal neuralgia [53].

Another common situation is represented by persistent idiopathic facial pain PIFP (atypical facial pain): a misdiagnosis of it is a common situation. In general, in all these cases, the important thing is to be able to distinguish between these two big families, in other words, to understand if the pain is due to a nerve lesion or to an odontogenic lesion.

## 4. Neuropathic Pain

The IASP describes as neuropathic the type of pain that is caused by a lesion or disease of the somatosensory nervous system [16,54,55]. This recent definition replaces the previous definition, which considered neuropathic pain as each form of pain “initiated or caused by a primary lesion or dysfunction or transitory perturbation in the peripheral or central nervous system” (Table 1) [21,56].

For a long time, the presence of pain following an injury to the nervous system has been traced back to different headings, such as nerve injury pain, neuralgia, deafferentation pain, neurogenic pain, and central pain. The last definition of IASP groups all these forms together.

As trivial as it may sound, there are changes in this new definition that cannot be overlooked. And this is because it points out that a general nervous dysfunction is no longer accepted as a sufficient criterion for the diagnosis; moreover, this new definition specifies that for it to be defined as neuropathic pain, the nervous lesion needs to affect the somatosensory system. This means that lesions or diseases outside the somatosensory pathways do not qualify the related pain as neuropathic [16,58].

Considering the orofacial region, we have seen how chronic forms of pain are not uncommon. But so far, we have not focused on exactly what kind of pain this chronic pain is that most patients with orofacial pain experience. And, in most cases, they are patients characterized by neuropathic pain.

Overall, the prevalence of neuropathic pain is not exactly known. Previous data showed an estimated percentage of 1–1.5% of the general population were affected by it [21]. Commonly, neuropathic pain has always been differentiated into two forms, peripheral and central neuropathic pain. Moreover, it is also important to consider that, very often, patients who present these forms of pain have a more complex background of systemic diseases, such as multiple sclerosis. Considering, for example, patients with a previous diagnosis of multiple sclerosis, 28% of them also present central neuropathic pain, the same for 75% of the patients who have already been diagnosed as affected by syringomyelia [21,59].

The causes that can lead to the onset of neuropathic pain are diverse: they can range from vascular compression, radiation, inflammation, trauma, infection, and exposure of the peripheral nervous system to neurotoxins can lead to pathologic damage [21,60,61]. It is critical to consider all the cases of neuropathic pain with an iatrogenic cause, following improper dental or oral surgery treatments.

### 4.1. Neuropathic Pain: Investigations

The concept of neuropathic pain is still an evolving concept. There are, therefore, no clearly defined diagnostic gold standards. There are some conditions in which the mechanism of nerve damage is very clear.

In these cases—such as post-herpetic neuralgia, painful diabetic neuropathy, and central post-stroke pain, for example—it can be difficult to recognize the condition sometimes, but once the diagnosis is made, it is not difficult to understand the underlying mechanism.

On the other hand, there are other conditions in which the presentation is not univocal and clear. It is, therefore, necessary to investigate and identify the nerve damage in order to define the pain as neuropathic [16].

Moreover, it is important to consider another point: when we focus on pain, in most cases, we are dealing with diseases and disorders in which most of the symptoms are subjective and in which the associated clinical signs are few or non-existent. It is also for this reason why it is difficult to define point-by-point diagnostic criteria [16].

Moreover, pain is a subjective sensation; for this reason, it could be useful to use questionnaires in order to assess and monitor the progression of the symptoms and the efficacy of therapy [62]. One frequently used solution is represented by different rating scales and questionnaires that have been developed to identify some discriminative features between neuropathic and non-neuropathic pain states, for example (Table 2).

Nowadays, an evaluation scheme is used. It was initially proposed in 2008, and it has been recently updated [16,63,64,65]. It recognizes three different types of neuropathic pain, namely: possible, probable, and definite neuropathic pain, distinguished according to the neurological history, the distribution of pain, and the presence and location of sensory signs, with an ending confirmatory test [16] (Table 3).

Clinically, it is possible to identify spontaneous neuropathic pain, which can be characterized by intermittent electric-shock-like pain paroxysms. These can be reported with an isolated presentation, but in some cases, it can also be associated with an ongoing pain. Most of the patients complain of burning, shooting, pricking, pins and needles, squeezing, or freezing pain [16,66]. Moreover, the sensation of pain could occur together with other non-painful abnormal sensations that can be both spontaneous and evoked. These additional sensations, in most cases, are represented by dysesthesia, an unpleasant abnormal sensation, and paraesthesia, an abnormal sensation that is not unpleasant.

Alongside spontaneous pain, many patients also complain of touch-evoked or cold-evoked pain, and, in most of the cases, sensation of allodynia (pain due to a stimulus that does not normally provoke pain) and hyperalgesia (increased pain from a stimulus that normally provokes pain) associated to mechanical and thermal stimuli could be found in patients with an already diagnosed sensory loss [16,17].

### 4.2. Pharmacological Treatment of Peripheral Neuropathic Pain: When and How to Treat?

The genesis of neuropathic pain is not unique, so it is difficult to define a therapeutic strategy that influences all forms of neuropathic pain.

In most cases, if a treatment strategy has been defined, it is based on drugs that are not first-line drugs for pain management: as mentioned, neuropathic pain is not typical pain with an inflammatory background [67]. On the contrary, it is characterized by a nerve involvement, requiring different drugs (Table 4 and Table 5). As dentists are quite often the first filter for the detection of pain, they must be informed about this option and aware of which kinds of drugs they are allowed to prescribe. The best option remains to make an initial differential diagnosis as soon as possible and, if neuropathic pain is suspected, to be prepared to refer the patient immediately to a specialist.

As indicated above, the interventional strategy led to the main scope of reducing or relieving headaches, sore muscles, arthritis, or other aches and pains. There are many different pain-killer drugs, and each one has pros and cons. Some types of pain respond better to certain treatments than others. Each person may also have a slightly different response to a pain reliever. Over-the-counter (OTC) medicines are good for many types of pain. There are two main types of OTC pain medicines: acetaminophen (Tylenol) and nonsteroidal anti-inflammatory drugs (NSAIDs). Aspirin, naproxen (Aleve), and ibuprofen (Advil, Motrin) are examples of OTC NSAIDs. If OTC medicines do not relieve pain, the doctor may prescribe something stronger or different [67].

Of all those that have been considered, the most successful drugs are represented by tricyclic antidepressants (TCA), gabapentin, pregabalin, and serotonin noradrenaline reuptake inhibitors (SNRI: duloxetine and venlafaxine). These are recommended as first-line drugs [16,68].

Then, there are other drugs with a weak recommendation. These included capsaicin 8% patches, lidocaine patches, and subcutaneous injections of botulinum toxin type A for peripheral neuropathic pain only [16,69].

Other authors have suggested that some effects may also be attributable to drugs such as tramadol and opioids, but these are treatments that are not recommended in long-term patient management [16].

The point is that, as effective as these lines of intervention could potentially be, the actual size and duration of the effect they have do not make them truly decisive in the treatment of the patient—if proposed as monotherapies. Moreover, safe and painless treatment is fundamental for a clinically practicing dentist, but recent data suggest greater effectiveness of a combined treatment [70].

For this reason, to complement purely classical drug therapy, alternative methods of intervention have developed over time: recently, research has been involved in investigating some unconventional approaches, such as hypnosis, acupuncture, electro-magnetic waves, ultrasound, and electro- and manual therapy [70,71,72,73,74,75].

#### Non-Pharmacological Interventions in the Treatment of Orofacial Pain

As mentioned above, the orofacial region can be affected by many different types of pain. Different forms of pain are characterized by a variety of modes of presentation, duration, population primarily affected, but also by a distinct underlying etiopathogenesis.

This diversity leads to the need to use different therapeutic strategies, but, at the same time, it also means that the treatment outcome is not always the same, and, as mentioned above, it cannot always be equally effective.

In recent years, therefore, new alternative methods have been proposed, in most cases, as a supportive therapy to the canonical drug treatment.

The first alternative that is increasingly being used is hypnosis. Actually, many medical conditions seem to respond better when drug treatment is combined with the practice of hypnosis [71]. Although this is an increasingly studied therapeutic strategy, it is still not entirely clear how it works. Recent data show that the use of hypnosis involves a modulation of brain activity, acting on some of the central nodes in the transmission of the pain stimulus—mainly on the primary and secondary somatosensory cortex, on the anterior cingulate cortex, on the mid-cingulate cortex, on the insula, and on the thalamus [71,76,77]. As mentioned earlier, interest in the role of hypnosis in pain management is widespread to different anatomical regions, but particular positive feedback has been found when hypnosis has been considered related to pain mainly focused on the dental/maxillofacial field, especially if the pain is associated with emergency situations, trauma, or acute inflammatory situations—all conditions in which the canonical drug therapies are contraindicated or, anyway, not so effective [70].

Another non-pharmacological intervention is represented by acupuncture. In this case, it shows different degrees of efficacy in a short period of time, even if data collected so far are not definitive, and, in most of the cases, they can only be considered as a starting point for further evaluations [73,78].

Moreover, next to oral acupuncture, abdominal acupuncture also has been considered in the last few years: it is represented by a relatively new microsystem technique that requires the insertion of needles at different depths at specific acupoints in the abdominal wall [72]. This type of non-pharmacological application is mainly used in the treatment of pain associated with temporomandibular dysfunction.

In the end, the most recent data are regarding the use of manual therapy in the management of orofacial pain, predominantly chronic pain associated with musculoskeletal dysfunctions, first and foremost, temporomandibular disorders [74,75].

## 5. Dentistry Pain: The Real Clinical Practice

In everyday practice, it is not uncommon for patients suffering from orofacial pain to go to their dentist asking for a solution. In our experience, general dentists are not often really well-informed about the difference between neuralgic and neuropathic pain, so they tend to treat all cases of pain in the same way: initially using NSAIDs and then, if these fail, with other dental treatments, like extractions, root canal treatments, etc. Patients themselves, who want the dentist to do something in the expectation that an action will have a benefit, often request treatment. As a result, and due to the fact that dentistry in many countries is mainly private, dentists want to keep the patient and avoid the risk of losing them for not treating them according to their wishes, so, consequently, they start a dental treatment even if it is not based on a real problem. Also, the pain sometimes has an iatrogenic origin, caused by surgical treatments that injured the nerve. In these cases, we need to understand which kind of lesion has been provoked: neuropraxia, axonotmesis, or neurotmesis; in some of those cases, the intervention from a microsurgeon can be considered (Table 6).

In trying to manage this important social problem and trying to provide proper support to dentists, we founded, in 2009, the SISDO (Italian Society for the Study of Pain) with the aim to provide general practitioners with practical support. Practically, with courses, lectures, articles, and the use of a website, we wanted to provide them initially with a correct guide for a proper differential diagnosis, having a correct check of the clinical dental situation. For these reasons, we also published the book: “Clinical, neurochemical and experimental aspects of orofacial pain in Dentistry” [79]. If, even after a proper consultation, X-rays, perio probe, etc., they cannot find relevant reasons for the patient’s pain from a dental point of view, they must be aware that the solution may be outside their area of expertise. In this case, SISDO (or similar societies with the same intent) will also provide them, if necessary, with a proper network of consultants (such as neurologists) who can tackle this problem using more appropriate tools. Of course, our expectation is that other countries will also consider this approach to support dentists in their daily work, providing patients with a more adequate solution to their pain. We do not expect dentists to be able to independently manage neuropathic pain; we have already considered in this paper how many tools are available now and how many will be available soon. Recently, specialists are also investigating possible treatments outside conventional approaches, like hypnosis, acupuncture, electromagnetic waves, and many others [70,71,72,73].

## 6. Conclusions

Following the IASP definition, pain can be defined as “an unpleasant sensory and emotional experience associated with actual or potential tissue damage, or terms of such damage”. The social influence of chronic or neuralgic pain is enormous, affecting quality of life, business ability, and money consumption, both for the individual and for society.

To properly recognize what type of pain is affecting our patients, and consequentially to initiate the most appropriate therapy to eliminate, or at least to reduce, their pain, will then be a very important task for doctors to improve the quality of life of our patients, reduce social costs, and increase their quality of life.

A better awareness among dentists, the very first filter of orofacial pain, will then be a task to be pursued to achieve better management of this kind of very invaliding problem.

## Figures and Tables

**Table 1:** Nociceptive, inflammatory, and neuropathic pain. A schematic summary of the main differences between nociceptive, inflammatory, and neuropathic pain.

Nociceptive Orofacial Pain	Inflammatory Orofacial Pain	Neuropathic Orofacial Pain
= normal nerve structure	= normal nerve structure	= ALTERED nerve structure
= associated with a physiological stimulation at the peripheral nerve that is transmitted by the components of the sensory trigeminal nerve	= noxious stimuli determine lesions in the tissue causing a local inflammation response and leading to the release of inflammatory mediators	= associated with nerve damages or injuries that lead to increased peripheral sensitization determining ectopic discharges and glia cell activation
= response to noxious stimulus leading to a **protective response**	= response to noxious stimulus with subsequent increase **in activity of peripheral nociceptors**	= response to non-noxious and noxious stimulation + manifestation of **spontaneous** pain not associated with any stimulation **due to ectopic discharges occurring in damaged nerves**

Adapted from Ref. [57].

**Table 2:** Pain evaluation systems. A schematic summary of the main different rating scales and questionnaires that are commonly used to evaluate and classified pain states.

Is it Possible to “Measure” the Pain?
VAS = Visual analogic scale
NRS = Numeric rating scale
VRS = Verbal rating scale
FPS = Faces pain scale (*children*)
EVENDOL = EValuation ENfant DOuLeur (*newborn*)
PIPP = Premature infant pain profile (*newborn*)
COMFORT neo scale (*post-surgery pain in children*)

**Table 3:** How to define neuropathic pain. It is possible to identify three different types of neuropathic pain: possible, probable, and definite neuropathic pain. These three forms can be distinguished according to the neurological history, the distribution of pain, and the presence and location of sensory signs, and they can be confirmed by an ending confirmatory test [16].

Neuropathic Pain	
Type	Main features
Possible	Previous relevant neurological lesion or disease; Pain distribution neuroanatomically plausible.
Probable	Pain associated with sensory signs in the same neuroanatomically plausible distribution on clinical examination.
Definite	Lesion or disease of the somatosensory nervous system confirmed by a diagnostic test.

Adapted from Ref. [16].

**Table 4:** Different types of pain, different treatment strategies. Pain in the orofacial region can be associated with several different causes, and it can be characterized by many different features and patterns of presentation. Consequently, it is not possible to think of a single therapeutic strategy; instead, targeted therapies with specific actions are needed, depending on the mechanism that is causing the pain itself.

Orofacial Pain Management	
Type of pain	Main drugs
Inflammatory pain	Anti-inflammatory drugs; Pain reliever drugs (e.g., paracetamol) [67].
Post-surgery pain	Anti-inflammatory drugs; Pain reliever drugs (e.g., paracetamol) [67].
Temporomandibular joint-associated pain	Anti-inflammatory drugs; Pain reliever drugs (e.g., paracetamol) [67].
Neuropathic pain	Opioids; Tricyclic antidepressants; Serotonin noradrenaline reuptake inhibitors.

**Table 5:** Nociceptive pain treatment. Schematic summary of the main drugs and therapeutic strategies that can be used in the management of nociceptive pain.

Nociceptive Pain Management	
Step	Main drugs
1—mild pain	**NON-opioid analgesics:** Acetaminophen; Aspirin; Other non-steroidal anti-inflammatory drugs.
2—moderate pain	**Moderate opioids:** Tramadol; Codeine; Oxycodone; (Associated with other non-opioid adjuvants).
3—severe pain	**Strong opioids:** Morphine; Levorphanol; Oxycodone.

**Table 6:** Different types of nerve injury. Neuropraxia, axonotmesis, or neurotmesis represent three different situations that dentists can face: the table summarizes the main characteristics of each condition.

Nerve Injuries			
Name	Definition	Functional recovery	Treatment
Neuropraxia	**Functional blackout of the nerve transmission and function caused by compression or stretching of the nerve.**	- Minimum of a few hours - Maximum of 12 weeks	- No treatment - Anti-inflammatory drugs
Axonotmesis—LEVEL 1	**Interruption of the axons with maintenance of the endoneuronal sheaths.**	- Nervous degeneration from the firs upstream Ranvier station or node - Regeneration rate = 1 mm/day - Approximately 1 year	- No treatment - Anti-inflammatory drugs
Axonotmesis—LEVEL 2	**Partial thickness scar of the endonerve.**	- More than 1 year	- Removal of the scar (=neurolysis) - Stumps suture (=neurorrhaphy)
Axonotmesis—LEVEL 3	**Full thickness scar of the endonerve.**	- Very difficult	- Removal of the scar (=neurolysis) - Stumps suture (=neurorrhaphy)
Neurotmesis	**Discontinuance of the sheaths (also epinerve) and of the axons.**	- Nonfunctional regeneration, if it occurs, leads to the formation of a neuroma	- Removal of the scar (=neurolysis) - Stumps suture (=neurorrhaphy)
